# Biometeorological Assessment of Mortality Related to Extreme Temperatures in Helsinki Region, Finland, 1972–2014

**DOI:** 10.3390/ijerph14080944

**Published:** 2017-08-22

**Authors:** Reija Ruuhela, Kirsti Jylhä, Timo Lanki, Pekka Tiittanen, Andreas Matzarakis

**Affiliations:** 1 Finnish Meteorological Institute, P.O. Box 503, FI-00101 Helsinki, Finland; kirsti.jylha@fmi.fi; 2 National Institute for Health and Welfare, P.O. Box 95, FI-70701 Kuopio, Finland; timo.lanki@thl.fi (T.L.); pekka.tiittanen@thl.fi (P.T.); 3 Research Center Human Biometeorology, German Meteorological Service, Stefan-Meier-Str. 4, D-79104 Freiburg, Germany; Andreas.Matzarakis@dwd.de

**Keywords:** mortality, thermal comfort index, heat stress, cold stress, climate, acclimatization

## Abstract

Climate change is expected to increase heat-related and decrease cold-related mortality. The extent of acclimatization of the population to gradually-changing thermal conditions is not well understood. We aimed to define the relationship between mortality and temperature extremes in different age groups in the Helsinki-Uusimaa hospital district in Southern Finland, and changes in sensitivity of the population to temperature extremes over the period of 1972–2014. Time series of mortality were made stationary with a method that utilizes 365-day Gaussian smoothing, removes trends and seasonality, and gives relative mortality as the result. We used generalized additive models to examine the association of relative mortality to physiologically equivalent temperature (PET) and to air temperature in the 43-year study period and in two 21-year long sub-periods (1972–1992 and 1994–2014). We calculated the mean values of relative mortality in percentile-based categories of thermal indices. Relative mortality increases more in the hot than in the cold tail of the thermal distribution. The increase is strongest among those aged 75 years and older, but is somewhat elevated even among those younger than 65 years. Above the 99th percentile of the PET distribution, the all-aged relative mortality decreased in time from 18.3 to 8.6%. Among those ≥75 years old, the decrease in relative mortality between the sub-periods were found to be above the 90th percentile. The dependence of relative mortality on cold extremes was negligible, except among those ≥75 years old, in the latter period. Thus, heat-related mortality is also remarkable in Finland, but the sensitivity to heat stress has decreased over the decades.

## 1. Introduction

Temperature dependence of mortality is often described as U-, V-, or J-shaped with increasing mortality towards both hot and cold temperature extremes, e.g., [1,2,3]. The shape of the mortality-temperature relationship and the temperature range of minimum mortality vary by latitude and climatic zone [4,5]. The optimal, minimum mortality temperature is lower in cold than in warm climates due to acclimatization of the population to their typical climatic conditions. In Finland, Northern Europe, the mortality is lowest when the daily mean temperature is in the range of 12–17 °C, while in Mediterranean countries the same is true at 22–25 °C [6,7,8].

Well-known past extreme events include the 2003 heat wave in Central and Western Europe, causing about 70,000 additional deaths [9], and the 2010 heat wave in Russia, leading to about 55,000 additional deaths [10]. These heat waves also extended to Finland, which has about 5.5 million inhabitants. According to Kollanus and Lanki [11], the number of non-accidental extra deaths was over 200 in 2003 and more than 300 in 2010. The impact of these two heat waves in Finland was most severe among the elderly, aged 75 years and older, as the increase in daily mortality was, on average, 21%. The most severe heat wave that has been studied in Finland took place in 1972 and caused about 800 extra deaths. After that, remarkable heat waves with increased mortality also occurred e.g., in 1973, 1978, 1988, 1995, and 1997 [7].

Most of the temperature-related mortality burden is contributable to cold in different climatic zones—even in tropical and sub-tropical areas [12]. The impacts of cold thermal conditions on mortality are more complex than the impacts of hot conditions due to different causal pathways. When the impacts of hot weather tend to appear with short lags, on the same day or within a couple of days, the increase in cold-related mortality can be found with some delay varying from days up to weeks [13,14,15]. On the other hand, Ebi and Mills [16] questioned the assumption that temperature is the reason for the strong seasonality with higher mortality in winter, particularly in case of cardiovascular, but also respiratory disease mortality.

Sensitivity to heat or cold stress varies according to many health and societal aspects, such as age, ill health, personal lifestyles, poverty, infrastructure and buildings, access to health care, and social structures [17,18]. Especially elderly and people with pre-existing medical conditions, such as cardio-vascular or respiratory diseases, diabetes, or chronic mental illnesses, are found to be vulnerable to temperature extremes [2,11,19,20]. Changes in sensitivity and adaptive capacity through, e.g., improved population health and the provision of health services may also change temperature-related mortality [2,18].

Short-term acclimatization to seasonal temperature variations takes place within a couple of weeks and should also be considered [21]. In the United States of America (USA), Lee et al. [22] found that heat effects were larger in the spring and early summer, and cold effects were larger in late fall. In addition, heat effects were larger in regions where high temperatures were less common, and vice versa for cold effects.

Long-term adaptation of the populations to their normal local climatic conditions takes place over decades through physiological acclimatization processes or through behavioural adaptation, such as housing or clothing suitable for the climate. According to Oudin Åström et al. [23] the minimum mortality temperature increased in Stockholm over the course of the 20th century, suggesting that autonomous adaptation took place in the Swedish population—in climatic conditions rather similar to those in Southern Finland. In another study, Oudin Åström et al. [24] found that the relative risk of mortality from cold and heat extremes remained stable over the period of 1980–2009.

Climate change is projected to increase heat-related mortality, especially due to more intense heat waves and a decrease in cold-related mortality due to fewer cold extremes [25]. Thus, global warming is expected to gradually alter the seasonal mortality cycle as well. Ballester et al. [3] assessed that in Europe, the rise in heat-related mortality would start to compensate for the reduction of deaths from cold during the second half of the century.

This long-term adaptation causes substantial uncertainty in projected changes in mortality and should be included in the assessments of climate change impacts on mortality. According to a review by Huang et al. [26], only half of the studies on projecting future heat-related mortality included acclimatization. For instance, Muthers et al. [27] concluded that heat-related mortality would increase significantly in Vienna until the end of the 21st century, also in an approach where long-term adaptation was included. On the other hand, Ballester et al. [3] suggested that if societies effectively adapt to a warmer climate, at the end of the century the total mortality due to thermal stress might decrease in Europe, when increases in heat-related mortality, decreases in cold-related mortality, and acclimatization are taken into account. Zacharias et al. [28] concluded that by the end of the 21st century, excess deaths due to ischemic heart diseases in Germany attributable to heat waves is expected to rise by factor 2.4 and 5.1 in the acclimatization and non-acclimatization approach, respectively.

Heat and cold stress depend on the energy exchange experienced by humans and are closely related to human thermoregulatory mechanisms and the human circulatory system [29,30,31,32]. Thermal indices based on the human energy balance require several meteorological input parameters: air temperature, air humidity, wind speed, and the short- and long-wave radiation fluxes, the total impact of the latter two being summarized as the mean radiant temperature [30,32].

A widely-used human biometeorological thermal index, the physiologically equivalent temperature (PET), is based on the human energy balance. It describes the temperature at a given place (outdoors or indoors) equivalent to the air temperature in a typical indoor setting with core and skin temperatures equal to those under the conditions being assessed. Units of PET, degrees Celsius, make the results comprehensible [32]. The standard PET values are valid for the assumed values of activity (80 W) and thermal resistance of the clothing (0.9) [31,33]. Moreover, the meteorological input parameters are to be measured at, or transferred to, the average height of a standing person’s gravity centre, 1.1 m above the ground [28,34].

Thermal indices such as PET describe the thermal environment of a person more realistically than a single parameter, such as outdoor air temperature [35]. However, it should be kept in mind that the meteorological input parameters for thermal indices often show remarkable temporal and spatial variability, and are therefore sources of large uncertainties in the indices. Wind speed and mean radiant temperature show the highest variability and are modified strongly by surroundings and obstacles, especially in complex urban areas [36]. Therefore, it is not self-evident that the use of energy balance-based rational thermal indices would always be the best choice for studies if the health impact data does not include precise spatial information. Quite often in studies on heat- and cold-related mortality, only outdoor air temperature is used as an explanatory variable.

The main aims of this study were (1) to quantify the increase in mortality related to both hot and cold temperature extremes according to different age groups in the Helsinki-Uusimaa hospital district in Southern Finland; (2) to study potential changes in time of the temperature-mortality relationships over the period of 1972–2014; and (3) to examine whether outdoor air temperature and PET give similar temperature-mortality relationships. Finally, we discussed the results from the viewpoint of potential acclimatization and changes in sensitivity of the Finnish population to temperature extremes.

## 2. Materials and Methods

### 2.1. Mortality Data in the Study Area

Daily numbers of all-cause deaths and annual population in the Helsinki-Uusimaa hospital district in Finland from 1971 to 2015 were obtained from Statistics Finland (Helsinki, Finland). The data included the number of all-aged deaths and the number of deaths in two age groups: 65–74 years and 75 years and older. From these data, we calculated the daily number of deaths in two additional age groups: younger than 65 years, and 65 years and older. Total number of deaths in the study period was 465,553, out of which 235,963 were 75 years and older, 98,967 were 65–74 years, and 130,623 were younger than 65 years. We interpolated daily population values from the annual population, and used them to calculate the daily mortality (1/100,000). Linear time trends for mortality with a 95% confidence level were determined in each age group.

The population in this hospital district (area 9.097 km^2^) increased from about 1.0 million to about 1.6 million during the study period. The age distribution during the study period changed remarkably as the share of elderly (≥65 years) increased from 9.1 to 16.5%. The life-expectancy in Finland over the study period has increased markedly. In 1980, the median age at death was 68.2 years for men and 75.4 years for women. In 2015, the corresponding values were 76.8 and 85.3 years, respectively [37].

### 2.2. Expected and Relative Mortality

**Figure 1:**
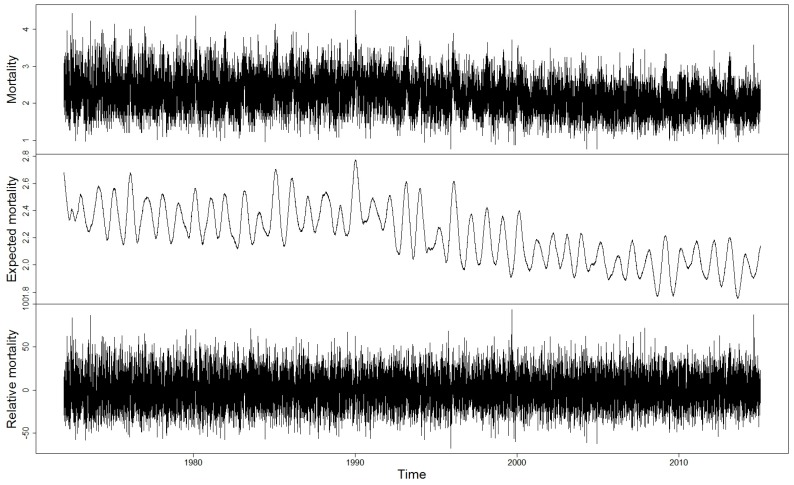
Time series of mortality (1/100,000) (upper panel), expected mortality (1/100,000) (middle), and relative mortality (%) (lower panel) in Helsinki-Uusimaa hospital district, 1972–2014. All-cause deaths and all age groups.

In order to study the impacts of temperature extremes on mortality and changes in these impacts over the decades, we applied the following method developed by Koppe and Jendritzky [21]. As a baseline mortality in each age group, we used expected mortality that was calculated for each day of the study period from the original mortality data using Gaussian smoothing with a filter of 365 days. The time series of expected mortality includes the seasonal cycle, but day-to-day variability is smoothed out (Figure 1, middle). Relative mortality is then the deviation of mortality from the expected mortality as a percentage. The time series of relative mortality exhibit day-to-day variability, whereas the seasonal cycle and long-term trends are removed (Figure 1, bottom).

The use of these stationary time series of relative mortality makes it possible to analyse impacts of temperature extremes over the decades regardless of the changes in population. The smoothing method shortens the time series of the mortality data from both ends. In the following, in comparisons of relative mortality to the meteorological data, we use full calendar years, thus 1972–2014.

### 2.3. Meteorological Data

Synoptic (three-hourly) temperature, relative humidity, wind speed, and global radiation data from the Helsinki-Vantaa weather station were used as input to calculate PET indices for the study period. We made the calculations with the RayMan model [38,39]. The daily mean values of PET were calculated from the three-hourly data to describe the daily average thermal conditions. While the emphasis of this paper is given to daily means of PET, we also calculated PET at 12 UTC (Universal Time Coordinated, at 15/14 local time during summer/winter) to describe the maximum daytime heat load and PET at 06 UTC (at 9/8 local time during summer/winter) to describe thermal conditions in the active morning hours.

In addition to the station-wise PET indices, we used spatial averages of daily mean air temperatures (T_avg_) over the Helsinki-Uusimaa hospital district as an explanatory variable for relative mortality. Values of T_avg_ were derived from the gridded 10 × 10 km dataset of the Finnish Meteorological Institute [40]. Spatial averages of daily mean maximum and minimum temperatures were calculated likewise.

The use of two indices, PET, based on station data, and T_avg_, based on gridded data, was motivated by the fact that weather conditions may vary remarkably within the Helsinki and Uusimaa province, especially depending on the distance from the Gulf of Finland and the wind direction, and the station-wise PET value may not describe thermal conditions in the hospital district. On the other hand, if both indices produce similar assessments of temperature-related mortality in our study area, confidence in the results is increased. Linear time trends with 95% confidence level were determined from the time series of the PET and T_avg_.

### 2.4. Assessing the Relationship between Relative Mortality and Thermal Indices

The impacts of the thermal conditions on the relative mortality in different age groups were studied by comparing the daily mean values of PET and T_avg_ with the mean value of relative mortality in the same and following day, thus applying a one-day lag. We used a generalized additive model (GAM) to describe the relationships between the relative mortality and the thermal indices. The exposure-response curves with 95% confidence intervals were fitted to the data by using penalized thin plate regression splines in the R package “mgcv” [41]. In addition to the whole study period, we conducted the analysis for two 21 year-long sub-periods, 1972–1992 and 1994–2014, in order to visualize potential changes in these relationships.

Another approach was used for quantitative analysis to assess relationships between relative mortality and the thermal indices: From the frequency distributions of daily values of PET and T_avg_, we determined the following percentiles: 1%, 2.5%, 5%, 10%, 25%, 50%, 75%, 90%, 95%, 97.5%, and 99% for the whole study period. These percentiles were used to classify the thermal indices into 12 categories. Then the mean values of relative mortality in these percentile categories were calculated with 95% confidence intervals for various age groups: all, <65, 65–74, ≥75, and ≥65 years.

In order to assess whether the relationships between relative mortality and the thermal indices have changed over the study period, we applied linear regression to explore linear time trends of the mean relative mortality in each percentile category and each age group for the whole study period. Furthermore, we concretized the changes in relative mortality by calculating the mean values of relative mortality in the two 21-year long sub-periods: 1972–1992 and 1994–2014 using the percentile categories that were defined from the whole study period. We tested the statistical significance of differences in the relative mortality between the sub-period percentile categories by Welch Two Sample *t*-test. The Shapiro-Wilk test was used to check the sub-period specific normality of the distributions of relative mortality.

The dependence between mortality and thermal conditions varies depending on the time window, thus, exposure time and lag, chosen for the study. The impacts of heat stress become apparent typically on a shorter time window than cold stress. In order to study impacts of prolonged heat waves or cold spells on mortality, we also calculated the relationships for longer time windows using 7- and 14-day averages of relative mortality and thermal indices without lag. These time windows also partly include delayed impacts and potential harvesting effects.

The statistical calculations were done with R version 3.2.3.

## 3. Results

### 3.1. Trends in the Thermal Indices and Mortality Data

Air temperature and PET have statistically significant increasing time trends (*p*-values < 0.001) indicating ongoing climate change. Regression coefficients for the linear time trends (with a 95% confidence level) in the period of 1972–2014 for PET daily mean value and daily mean temperature in Helsinki-Vantaa were 0.37 °C/decade (0.22, 0.52) and 0.44 °C/decade (0.33, 0.55), respectively. Based on the gridded data, the trend for the daily mean temperature as an average over the hospital district was 0.36 °C/decade (0.24, 0.47).

Lengthening lifetime can be seen as statistically significant (*p*-values < 0.001) decreasing mortality trends in all age groups in the Helsinki-Uusimaa hospital district. The all-aged mortality (1/100,000) was 2.2 (±0.5) on average during the study period, with a decreasing trend of −0.116/decade (−0.122, −0.110). The mortality among 75 years and older during the study period was on average 24 ± 8 and the decreasing trend in mortality was clear, −2.1/decade (−2.2, −2.0). In the relative mortality, there are no time trends, because the methodology to calculate relative mortality removes trends from the time series, as explained in the Methods section.

### 3.2. Relationships between Relative Mortality and Thermal Indices

In the following, we first visually inspect how daily values of relative mortality, i.e., the deviations from expected mortality values, are related to two thermal indices, PET and the spatially-averaged temperature (T_avg_). Means, ranges, and temporal trends of relative mortality values are then studied as a function of percentiles of the indices.

**Figure 2:**
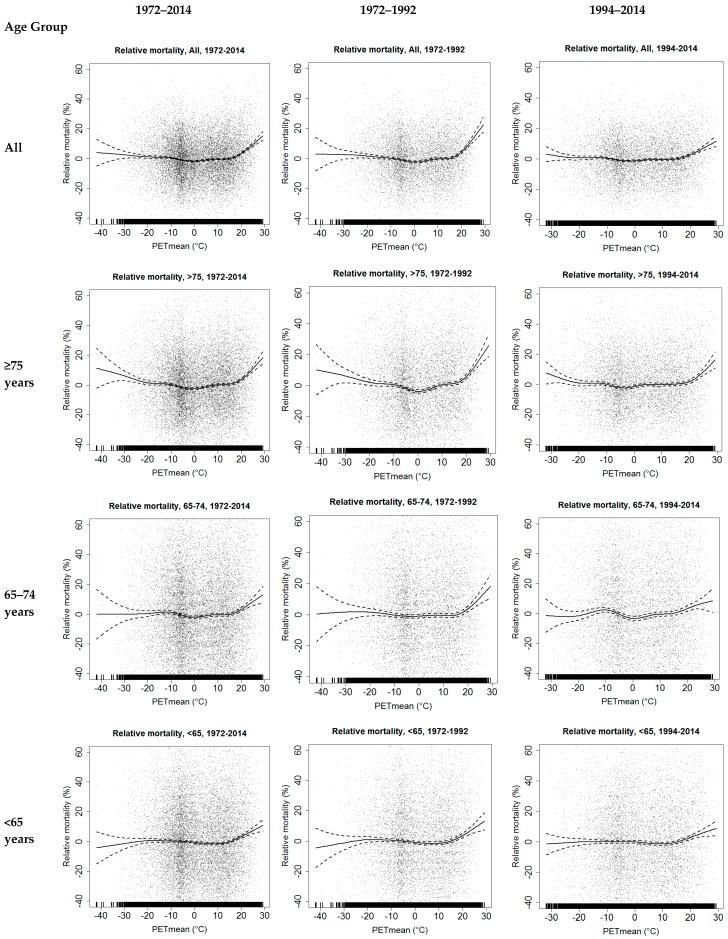
Scatterplots of relative mortality in different age groups and the daily mean value of physiologically equivalent temperature (PET) in the Helsinki-Vantaa weather station in the period of 1972–2014 and in two 21-year time periods, 1972–1992 and 1994–2014. Generalized additive models (GAM) fitted to the data (solid curves) with 95% confidence intervals (dashed curves).

The dependencies of the two-day mean of relative mortality on the daily mean value of PET in the whole study period (1972–2014) and in the two 21-year sub-periods (1972–1992 and 1994–2014) according to different age groups are presented in Figure 2. The modelled relative mortality-PET association curves show a clear increase from the expected mortality in all age groups at the PET mean value exceeding 20 °C. The slope is steepest in the age group ≥75 years. The increase in relative mortality is less in the cold than in the hot extreme conditions. In the cold thermal range, a slight increase in relative mortality with decreasing PET is seen only in the age group ≥75 years at the PET values below −30 °C. Some changes in time in the modelled relative mortality-PET association curves can be seen in both cold and hot extremes. In the latter sub-period, the increase in relative mortality with high values of PET is still apparent in all age groups, but smaller than previously. Moreover, in the latter sub-period, the dependence of relative mortality on PET in the cold thermal range is negligible.

**Figure 3:**
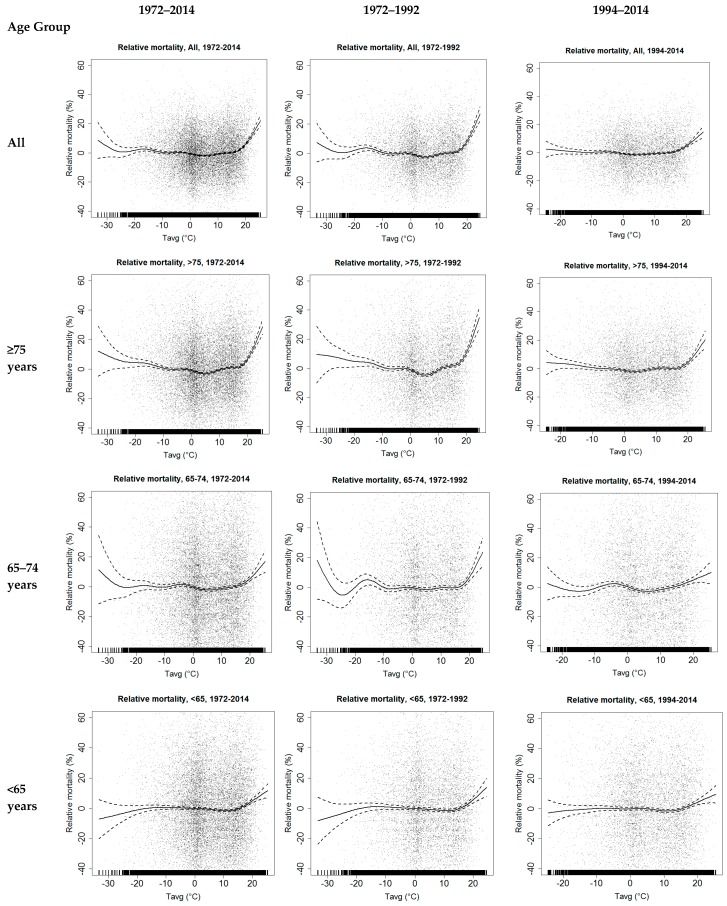
Scatterplots of relative mortality in different age groups and the daily mean temperature (T_avg_) in the Helsinki-Uusimaa hospital district in the period of 1972–2014 and in two 21-year time periods, 1972–1992 and 1994–2014. GAM fitted to the data (solid curves) with 95% confidence intervals (dashed curves).

The relationship between relative mortality and spatially-averaged daily mean temperature in the hospital district shows similar characteristics to the dependence of relative mortality on PET, especially in the hot extreme temperature range (Figure 3). However, in the cold temperature range, the T_avg_ dependencies differ somewhat from the PET dependencies: especially in the age group 65–74 years, the relative mortality-T_avg_ relationship in the cold range shows increases and decreases that are not seen in the relative mortality-PET relationship.

Relative mortality mean values in different percentile categories of daily mean PET and T_avg_ distributions are presented in Table 1 and Table 2, respectively, for different age groups. In the data covering the whole study period of 1972–2014, in the hot extreme end of the PET and T_avg_ distributions, the relative mortality values are elevated, by more than about 4% in the percentiles above 95%. The highest increase in mortality, 18.6%, was found above the 99th percentile of the T_avg_ distribution among the elderly, 75 years and older, but even among the younger population (<65 years) the increase in mortality was almost 10% (Table 2). The increase in relative mortality in the highest percentile category appears to be larger for T_avg_ than for PET.

The increase of the relative mortality at the cold extreme end of the distributions is smaller than in the hot extreme, typically only a few percent in the time period of 1972–2014. The largest deviation from the expected mortality, 5.2%, was found in the first percentile category of the PET distribution (below −25.1 °C) among the elderly, 75 years and older (Table 1).

Although the original time series of relative mortality by definition do not have long-term trends in time, this is not necessarily true when relative mortality values in different percentile categories of thermal indices are considered. Statistically significant decreasing linear trends in the relative mortality were indeed found in the highest percentile categories, above the 99th percentile of PET and T_avg_ in all age groups, except among those 65–74 years old. In the age group ≥75 years, the decreasing trend is seen also in more common thermal conditions, in PET and T_avg_ percentile categories above 90%. In the cold thermal range, only two statistically significant decreasing trends were found.

The considerations of the relative mortality in the various percentile categories of PET and T_avg_ show clear differences between the two 21-year sub-periods (Table 1 and Table 2, last columns). Above the 99th percentile of the PET distribution, a decrease of about 10%, from 18.3% in the period of 1972–1992 to 8.6% in 1994–2014, was found in all-aged relative mortality (Table 1). In the temperature distribution, the decrease was even greater, about 12%, from 22.5 to 10.7%, respectively (Table 2). In the age group ≥75 years, the decreases between the two sub-periods in the percentiles above the 90th were clear, although they did not reach the level of statistical significance in either case. As an example, the mean relative mortality was equal in the PET percentile class of 90 to 95th of the former sub-period and the class 97.5 to 99th of the latter sub-period. It is also noteworthy that the increase in relative mortality almost disappeared between the 90th to 97.5th percentile of both PET and T_avg_ distributions in the latter sub-period (Table 1 and Table 2). Among the group aged <65 years, the decrease in time in relative mortality between the sub-periods above the 99th percentile was 11–12%, from 15.7 to 4.0% for PET (Table 1), and from 16.3 to 5.6% for T_avg_ (Table 2).

In the cold thermal range, the changes in relative mortality between the two sub-periods were small and inconsistent (Table 1 and Table 2). However, in the latter sub-period, the dependence of relative mortality on cold extremes almost disappeared, except among those aged 75 years and older. In general, the changes between the two sub-periods were more consistent in the PET percentiles than in the T_avg_ percentiles, and more consistent in the warm thermal range than in the cold thermal range.

In addition to the narrow time window of two days for mortality, considered above, we studied the effect of the length of the time window on the outcomes by also making calculations using 7- and 14-day averages of mortality and explaining the thermal indices, without lag. The dependencies of relative mortality on PET (Supplementary Material, Figures S1 and S2 and Tables S1 and S2) and T_avg_ are also J-shaped in these time windows, indicating that the impact of heat stress on relative mortality is more pronounced than the impact of cold stress. In the longer time windows, the differences between the sub-periods become statistically significant in a larger number of percentile categories than in the two-day time window. Hence, the calculations in these time windows confirm our previous finding that the tendency of hot extremes to increase mortality has somewhat weakened over the decades. Furthermore, the outcomes in the longer time windows also suggest a decrease in the impact of the coldest extreme conditions on mortality but, interestingly, also a small increase in the more common wintertime thermal range with PET values around −10 °C among the group aged 65–74 years.

In this paper, we report the dependencies of relative mortality on daily mean values of PET and T_avg_. However, we made similar calculations also using PET at 12 and 06 UTC and daily maximum and minimum temperatures. Numerically, the relationships are somewhat different (data not shown) compared to the calculations with daily mean values, but the generic outcomes—increases in mortality in the highest percentiles of the thermal range, and decreasing impacts of heat and cold stress from the first to the later sub-period—are the same as when using daily mean values of thermal indices.

## 4. Discussion

Our study shows that the impact of heat stress on mortality is remarkable in the climate of Finland, in Northern Europe. The increase in relative mortality appears above the 95th percentile of the thermal distribution and is highest above the 99th percentile among those 75 years and older. The increase in relative mortality is greater in hot than in cold extreme conditions. This outcome may seem to contradict the common understanding that cold-related mortality is more remarkable than heat-related mortality (e.g., [7]). However, this difference is due to the method used in our study. Instead of absolute mortality we used relative mortality, defined as the deviation from the expected mortality, which includes seasonal variation. Thus, we studied the weather dependence of the mortality deviation from its expected seasonal value. According to our study, this method, developed by Koppe and Jendritzky [21], to calculate relative mortality is feasible especially when assessing impacts of temperature extremes. The method has also been applied, e.g., by Muthers et al. [27], in mortality studies in Vienna, and by Zacharias et al. [28] for ischemic heart diseases in Germany.

The temperature dependence of mortality in the cold side of the thermal distribution is more complex than in the warm side, and might require further studies with more sophisticated methods, such as a distributed lag model between mortality and thermal conditions, e.g., [12,42]. The cold-related mortality may also be related to the behaviour of people and exposure to the cold stress. Based on a population study in Finland, people spent only 4% of their total time under cold exposure, most of it (71%) during leisure time [43]. In extremely cold conditions, people avoid outdoor activities and are not exposed to cold stress for a long period of time, while in more typical cold conditions they may spend more time outdoors and are therefore more exposed to the cold stress. Interestingly, we found also a small increase in sensitivity in the thermal range with PET values around −10 °C among the group aged 65–74 years that might be worth further studies. In Finland, the indoor conditions are also typically thermally comfortable in the cold season due to the heating of houses. On the other hand, the cooling of houses in summer is currently rare in Finland, especially in older buildings. The heat stress inside buildings during heat waves may be severe depending on the building characteristics and intensity of the urban heat island (e.g., [44,45]).

The time series of relative mortality in our study are stationary, i.e., without a seasonal cycle or a trend in time. The use of the concept of relative mortality enables us to study changes in the temperature dependence of mortality during the study period. Our study shows a decrease in the impact of hot extremes on mortality in all age groups over four decades. This suggests that Finns, in general, have become less sensitive to very warm weather than previously. Thus, our study in the Finnish population supports the findings of some other studies on decreasing sensitivity to temperature extremes, e.g., [42,46]. Our study also confirms the earlier studies of Donaldson et al. [47] and Näyhä [7], which conclude that heat-related mortality has decreased in Finland over decades. On the other hand, de’Donato et al. [48] reported a reduction in heat-related mortality in some European cities, but suggested an increasing heat effect in Helsinki and Stockholm. However, in that research two quite short time periods were compared, namely 1996–2002 and 2004–2010. Additionally, the latter period included one of the most significant heat waves in Finland in summer 2010.

In studies related to acclimatization or changes in sensitivity to temperature extremes, sufficiently long time series, e.g., in our study over 40 years, should be used to capture inter-annual climate variability and any potential long-term climatic and non-climatic trends. On the other hand, a time period of about 20 years may be adequate, if the goal is to estimate impacts of extreme temperatures on mortality in the current climate and population structure. In our study, the increase in relative mortality with increasing temperature appeared to be larger when assessed in the whole study period than when assessed in only the latter sub-period. As we can see in Figure 1, the level of expected mortality in the Helsinki-Uusimaa hospital district has been on a fairly stable level since 2000. Accordingly, a time period starting around 2000 could be sufficiently long for health impact assessments in the current climate of Finland.

Based on our results, the dependence of mortality on extreme temperatures has weakened in time, even among those 75 years and older. Nonetheless, it is beyond the scope of the present paper to firmly attribute the decreased temperature-related mortality burden to any physiological or behavioural changes of humans or infrastructural changes in society, such as improved public health or lengthening of life time. While our study strongly suggests that the sensitivity of the Finnish population to temperature extremes has decreased in the past decades, it does not provide new information about long-term acclimatization. Our study did not capture changes in minimum mortality temperature, which would have been an important indicator for acclimatization as, e.g., in studies of Oudin Åström et al. [23] and Ballester et al. [3]. However, the decreasing impact of hot extremes on mortality that we found in our study might partly be due to long-term acclimatization as well.

In northern countries like Finland, the possibilities for future acclimatization to gradually increasing mean temperatures are good, since the limits of physiological acclimatization are not a threat, as in many parts of the world in hot climates. However, heat waves are expected to also become more frequent and intensive in Finland [49], and therefore planned adaptation measures to minimize heat-related health risks should not be neglected.

A limitation in our research was that the dataset did not include information on causes of death. Hence, we were not able to investigate whether heat- and cold-related mortality decreased among temperature-sensitive risk groups, such as people with heart and respiratory illnesses, or whether the decrease can be explained mainly by a healthier population in general. However, the recent research of Ryti et al. [50] on cold spells and sudden cardiac deaths suggests that the use of cardioprotective medication decreases the risk of sudden death during cold spells.

An important motivation for our study was to provide information for assessing impacts of climate change on mortality. Since potential acclimatization to gradually-warming climate and changes in sensitivity of the population to temperature extremes are important sources of uncertainties in studies related to climate change impacts on temperature-related mortality, there is a clear need for multi-disciplinary research.

One of our aims was to study the feasibility of different biometeorological indices to describe the dependence of mortality on thermal conditions. Meteorological input variables to calculate physiologically equivalent temperature, PET, are air temperature, humidity, wind speed, and solar radiation. Therefore, PET describes the thermal environment of a person more realistically than air temperature alone. In our study, PET seemed to provide a more consistent mortality-thermal condition relationship than air temperature throughout the thermal distribution, especially in the cold thermal range. Morabito et al. [51] also concluded that indices, which account for the extra effect of wind speed and solar radiation, fit best with all-cause elderly mortality.

The calculation of PET values requires sub-daily meteorological data and its spatial distribution is affected remarkably by topography and the built environment. Here, we also reported the mortality dependence on spatially-averaged gridded temperature data. Based on our results, temperature may give results that are in many cases good enough for studies related to weather and climate change impacts on mortality, at least in the warm thermal range. The use of gridded temperature data instead of station-wise meteorological data may, e.g., extend the possibilities to study weather impacts on mortality in sparsely-populated larger areas, which is the case in most parts of Finland and in areas having a sparse weather station network. Furthermore, in climate change impact studies, it may be adequate to use temperature scenarios alone, without projections for all the input variables needed for calculations of PET. Compared to inaccuracies arising from the use of temperature instead of PET, the other, non-climatic sources of uncertainty, discussed above, may be more influential.

The Finnish Meteorological Institute started to issue heat wave and cold spell warnings in 2011 but, so far, they have not been fully utilized in the health sector. Our findings on decreasing sensitivity to temperature extremes over the decades in Finland show the importance of the work to improve public health, and of well-functioning healthcare in the prevention of heat-related mortality and adaptation to climate change.

## 5. Conclusions

Heat-related mortality is also remarkable in northern countries such as Finland. The increase of the relative mortality in the hot extreme of the thermal distributions is more than in the cold extreme. On the other hand, from the 43-year long time series of mortality and meteorological data, we found a statistically significant decrease in relative mortality in upper percentiles of the thermal distribution, indicating a decreasing sensitivity to heat stress over the decades.

Both PET and spatially-averaged temperature are feasible indicators in modelling the dependence of mortality on thermal environment, depending on the scope of the study and availability of the data.

Further studies to elaborate the dependence of mortality on thermal conditions would require modelling of delayed effects by applying, e.g., distributed lag non-linear models. In studies on acclimatization, long time series of mortality should be used; according to our study, a period of at least several decades is needed to find time trends in sensitivity.

## Figures and Tables

**Table 1:** Mean relative mortality (95% CI) of different age groups in percentiles of daily mean values of physiologically equivalent temperature (PET)at the Helsinki-Vantaa airport, and their linear time trends in the period of 1972–2014 with statistical significance. Mean relative mortality in two 21-year sub-periods, 1972–1992 and 1994–2014, and the statistical significance of the differences in relative mortality between the sub-periods.

Percentiles	PET Range	Relative Mortality 1972–2014 (%)		Trend (%/10 years)		*t*-Test	Relative Mortality 1972–1992 (%)		Relative Mortality 1994–2014 (%)		*t*-Test
All											
0–1	−42.0, −25.1	2.1	(−0.2, 4.4)	0.3	(−1.6, 2.2)		2.5	(−0.6, 5.6)	1.7	(−1.7, 5.1)	
1–2.5	−25.1, −20.9	1.9	(0.2, 3.6)	−0.6	(−2.0, 0.9)		2.7	(0.3, 5.0)	0.7	(−2.0, 3.3)	
2.5–5	−20.9, −17.1	0.9	(−0.5, 2.4)	−0.9	(−2.1, 0.2)		2.4	(0.4, 4.3)	−0.4	(−2.5, 1.7)	
5–10	−17.1, −12.8	0.2	(−0.8, 1.2)	0.5	(−0.3, 1.3)		−0.4	(−1.8, 1.0)	0.9	(−0.7, 2.4)	
10–25	−12.8, −6.9	0.4	(−0.2, 0.9)	0.1	(−0.3, 0.6)		0.3	(−0.5, 1.2)	0.4	(−0.4, 1.2)	
25–50	−6.9, 0.4	−1.5	(−1.9, −1.1)	0.1	(−0.2, 0.5)		−1.5	(−2.2, −0.9)	−1.6	(−2.2, −1.0)	
50–75	0.4, 11.3	−0.9	(−1.4, −0.5)	0.3	(−0.1, 0.6)		−1.3	(−1.9, −0.7)	−0.6	(−1.2, −0.1)	
75–90	11.3, 17.3	0.0	(−0.6, 0.6)	−0.3	(−0.8, 0.2)		0.3	(−0.6, 1.2)	−0.2	(−1.0, 0.6)	
90–95	17.3, 20.4	2.5	(1.5, 3.6)	−0.4	(−1.2, 0.4)		3.1	(1.6, 4.7)	2.1	(0.8, 3.4)	
95–97.5	20.4, 22.6	4.4	(2.9, 5.9)	−1.0	(−2.2, 0.2)		5.2	(3.1, 7.4)	3.5	(1.4, 5.6)	
97.5–99	22.6, 24.5	7.2	(5.2, 9.3)	−1.5	(−3.2, 0.1)		9.5	(6.3, 12.8)	5.9	(3.2, 8.5)	
99–100	24.5, 29.4	11.7	(8.9, 14.5)	−3.9	(−6.0, −1.8)	***	18.3	(12.4, 24.3)	8.6	(5.6, 11.5)	**
Aged ≥75 years											
0–1	−42.0, −25.1	5.2	(2.5, 7.9)	−0.4	(−2.7, 1.8)		5.7	(2.0, 9.3)	4.7	(0.6, 8.7)	
1–2.5	−25.1, −20.9	2.1	(−0.3, 4.5)	0.3	(−1.7, 2.2)		2.1	(−1.3, 5.4)	1.8	(−1.7, 5.2)	
2.5–5	−20.9, −17.1	2.6	(0.7, 4.5)	−2.0	(−3.5, −0.5)	*	4.9	(2.1, 7.7)	0.4	(−2.2, 3.0)	*
5–10	−17.1, −12.8	0.0	(−1.5, 1.4)	0.6	(−0.6, 1.7)		−0.9	(−2.9, 1.2)	0.9	(−1.1, 3.0)	
10–25	−12.8, −6.9	0.1	(−0.7, 0.9)	0.2	(−0.5, 0.9)		0.3	(−1.0, 1.6)	0.0	(−1.0, 1.1)	
25–50	−6.9, 0.4	−2.3	(−2.9, −1.7)	0.3	(−0.2, 0.8)		−2.5	(−3.5, −1.6)	−2.2	(−3.0, −1.4)	
50–75	0.4, 11.3	−0.8	(−1.4, −0.2)	0.6	(0.1, 1.1)	*	−1.6	(−2.5, −0.6)	−0.1	(−0.9, 0.79	*
75–90	11.3, 17.3	0.8	(0.0, 1.6)	−0.4	(−1.0, 0.3)		1.4	(0.1, 2.7)	0.3	(−0.8, 1.4)	
90–95	17.3, 20.4	3.1	(1.6, 4.6)	−1.3	(−2.5, −0.2)	*	4.7	(2.2, 7.2)	1.9	(0.1, 3.7)	
95–97.5	20.4, 22.6	4.0	(1.9, 6.1)	−2.3	(−4.0, −0.6)	**	6.0	(2.7, 9.3)	1.8	(−1.0, 4.5)	*
97.5–99	22.6, 24.5	7.9	(4.7, 11.1)	−3.6	(−6.1, −1.1)	**	13.4	(7.6, 19.3)	4.6	(0.9, 8.3)	*
99–100	24.5, 29.4	14.3	(10.4, 18.3)	−4.8	(−7.8, −1.9)	**	21.0	(12.3, 29.7)	11.1	(7.1, 15.2)	*
Aged 65–74 years											
0–1	−42.0, −25.1	−0.7	(−5.3, 3.9)	−1.6	(−5.5, 2.3)		1.2	(−4.8, 7.2)	−2.9	(−10.2, 4.4)	
1–2.5	−25.1, −20.9	2.4	(−1.5, 6.3)	−2.4	(−5.6, 0.8)		4.7	(−0.2, 9.7)	−0.2	(−6.4, 6.1)	
2.5–5	−20.9, −17.1	−1.6	(−4.4, 1.1)	−0.4	(−2.6, 1.8)		0.0	(−4.0, 4.0)	−3.2	(−7.0, 0.7)	
5–10	−17.1, −12.8	0.1	(−1.9, 2.1)	0.3	(−1.3, 1.9)		0.0	(−2.7, 2.7)	−0.2	(−3.1, 2.7)	
10–25	−12.8, −6.9	1.8	(0.6, 2.9)	0.8	(−0.2, 1.7)		0.9	(−0.7, 2.6)	2.7	(1.0, 4.5)	
25–50	−6.9, 0.4	−1.6	(−2.5, −0.7)	0.3	(−0.5, 1.0)		−1.6	(−2.9, −0.4)	−1.5	(−2.9, −0.1)	
50–75	0.4, 11.3	−1.0	(−1.9, 0.0)	−0.5	(−1.3, 0.2)		−0.6	(−1.9, 0.7)	−1.5	(−2.9, −0.1)	
75–90	11.3, 17.3	−0.6	(−1.8, 0.7)	−0.2	(−1.2, 0.8)		−0.4	(−2.2, 1.3)	−0.7	(−2.5, 1.1)	
90–95	17.3, 20.4	3.1	(0.9, 5.2)	0.7	(−1.0, 2.4)		2.5	(−0.6, 5.5)	3.9	(0.8, 7.0)	
95–97.5	20.4, 22.6	4.7	(1.6, 7.8)	−0.2	(−2.7, 2.3)		5.3	(1.4, 9.1)	4.5	(−0.2, 9.3)	
97.5–99	22.6, 24.5	6.7	(2.5, 10.8)	0.3	(−3.0, 3.6)		7.2	(0.9, 13.5)	6.0	(0.5, 11.6)	
99–100	24.5, 29.4	10.9	(5.9, 15.9)	−3.7	(−7.5, 0.1)		17.7	(7.9, 27.4)	7.7	(2.0, 13.4)	
Aged <65 years											
0–1	−42.0, −25.1	−1.6	(−6.2, 3.0)	2.4	(−1.4, 6.3)		−1.7	(−8.0, 4.7)	−1.5	(−8.2, 5.3)	
1–2.5	−25.1, −20.9	0.6	(−2.8, 4.0)	−1.0	(−3.9, 1.8)		1.9	(−2.5, 6.2)	−1.6	(−7.0, 3.8)	
2.5–5	−20.9, −17.1	0.6	(−1.8, 3.0)	−0.1	(−2.1, 1.8)		1.1	(−2.1, 4.3)	0.1	(−3.7, 3.8)	
5–10	−17.1, −12.8	0.8	(−1.1, 2.6)	0.7	(−0.7, 2.2)		0.2	(−2.2, 2.6)	1.6	(−1.2, 4.5)	
10–25	−12.8, −6.9	−0.1	(−1.1, 0.9)	−0.5	(−1.3, 0.3)		0.0	(−1.5, 1.4)	−0.7	(−2.1, 0.7)	
25–50	−6.9, 0.4	0.0	(−0.8, 0.8)	0.1	(−0.5, 0.8)		0.0	(−1.2, 1.1)	0.1	(−1.2, 1.3)	
50–75	0.4, 11.3	−1.5	(−2.3, −0.7)	0.4	(−0.3, 1.0)		−1.5	(−2.7, −0.4)	−1.2	(−2.3, 0.0)	
75–90	11.3, 17.3	−0.8	(−1.8, 0.3)	−0.5	(−1.4, 0.3)		−0.7	(−2.2, 0.8)	−1.1	(−2.6, 0.4)	
90–95	17.3, 20.4	1.7	(−0.1, 3.5)	−0.1	(−1.5, 1.4)		1.7	(−1.0, 4.4)	1.3	(−1.2, 3.8)	
95–97.5	20.4, 22.6	5.6	(2.7, 8.4)	0.0	(−2.3, 2.3)		5.0	(1.0, 9.0)	6.0	(2.1, 10.0)	
97.5–99	22.6, 24.5	7.0	(3.7, 10.4)	0.3	(−2.4, 2.9)		5.8	(1.5, 10.1)	7.9	(3.1, 12.6)	
99–100	24.5, 29.4	7.7	(3.7, 11.7)	−3.1	(−6.2, −0.1)	*	15.7	(8.4, 22.9)	4.0	(−0.7, 8.7)	**

* *p* < 0.05, ** *p* < 0.01, *** *p* < 0.001.

**Table 2:** Mean relative mortality of different age groups (95% CI) in percentiles of daily mean temperature on average in the Helsinki-Uusimaa hospital district (T_avg_), and time trends in the period of 1972–2014, and two 21-year sub-periods: 1972–1992 and 1994–2014.

Percentiles	T_avg_ Range	Relative Mortality 1972–2014 (%)		Trend (%/10 years)		*t*-Test	Relative Mortality 1972–1992 (%)		Relative Mortality 1994–2014 (%)		*t*-Test
All											
0–1	−33.4, −18.5	0.5	(−1.7, 2.8)	−0.3	(−2.2, 1.6)		1.5	(−1.5, 4.5)	−0.9	(−4.3, 2.5)	
1–2.5	−18.5, −14.1	3.2	(1.3, 5.0)	−0.7	(−2.2, 0.8)		3.3	(0.8, 5.8)	2.6	(−0.1, 5.3)	
2.5–5	−14.1, −10.7	1.7	(0.2, 3.1)	−0.8	(−1.9, 0.4)		2.8	(1.0, 4.7)	0.2	(−2.2, 2.5)	
5–10	−10.7, −6.6	−0.2	(−1.2, 0.8)	0.3	(−0.5, 1.2)		−0.2	(−1.6, 1.1)	0.0	(−1.5, 1.6)	
10–25	−6.6, −0.8	0.3	(−0.1, 1.0)	0.2	(−0.2, 0.7)		0.1	(−0.8, 0.9)	0.5	(−0.3, 1.3)	
25–50	−0.8, 4.8	−1.4	(−1.7, −0.9)	0.2	(−0.2, 0.5)		−1.4	(−2.0, −0.8)	−1.4	(−2.0, −0.8)	
50–75	4.8, 12.7	−1.1	(−1.6, −0.7)	0.2	(−0.2, 0.5)		−1.5	(−2.2, −0.9)	−0.8	(−1.4, −0.2)	
75–90	12.7, 16.7	0.3	(−0.3, 0.9)	−0.1	(−0.6, 0.4)		0.5	(−0.4, 1.4)	0.2	(−0.6, 1.0)	
90–95	16.7, 18.6	1.7	(0.8, 2.7)	−0.5	(−1.2, 0.3)		2.4	(1.0, 3.9)	1.1	(−0.2, 2.4)	
95–97.5	18.6, 19.9	4.3	(2.8, 5.8)	−1.5	(−2.7, −0.3)	*	6.0	(3.7, 8.3)	2.6	(0.6, 4.7)	*
97.5–99	19.9, 21.4	5.6	(3.5, 7.6)	0.4	(−1.2, 2.0)		4.6	(1.3, 7.9)	6.1	(3.5, 8.6)	
99–100	21.4, 25.4	15.2	(12.5, 18.0)	−4.1	(−6.0, −2.3)	***	22.5	(17.7, 27.3)	10.7	(7.7, 13.8)	***
Aged ≥75 years											
0–1	−33.4, −18.5	3.9	(1.1, 6.8)	−2.0	(−4.4, 0.4)		5.9	(2.0, 9.8)	0.9	(−3.1, 5.0)	
1–2.5	−18.5, −14.1	4.1	(1.5, 6.7)	0.1	(−2.0, 2.2)		3.5	(−0.2, 7.2)	4.5	(0.9, 8.1)	
2.5–5	−14.1, −10.7	3.3	(1.4, 5.3)	−0.8	(−2.4, 0.7)		4.4	(1.8, 7.0)	2.0	(−0.9, 4.9)	
5–10	−10.7, −6.6	−0.8	(−2.2, 0.6)	0.3	(−0.9, 1.4)		−0.9	(−2.9, 1.1)	−0.3	(−2.3, 1.7)	
10–25	−6.6, −0.8	0.0	(−0.8, 0.9)	0.2	(−0.5, 0.8)		0.0	(−1.3, 1.3)	0.1	(−1.0, 1.1)	
25–50	−0.8, 4.8	−2.0	(−2.6, −1.4)	0.3	(−0.2, 0.8)		−2.2	(−3.1, −1.2)	−1.9	(−2.7, −1.1)	
50–75	4.8, 12.7	−1.3	(−1.9, −0.7)	0.7	(0.2, 1.2)	**	−2.3	(−3.3, −1.4)	−0.3	(−1.1, 0.5)	
75–90	12.7, 16.7	1.0	(0.2, 1.8)	−0.2	(−0.9, 0.4)		1.7	(0.4, 3.0)	0.5	(−0.6, 1.6)	
90–95	16.7, 18.6	2.2	(0.8, 3.6)	−1.2	(−2.2, −0.1)	*	3.7	(1.5, 6.0)	0.8	(−0.9, 2.5)	*
95–97.5	18.6, 19.9	4.7	(2.5, 6.9)	−3.3	(−5.0, −1.6)	***	8.2	(4.4, 11.9)	1.6	(−1.0, 4.2)	**
97.5–99	19.9, 21.4	5.7	(2.8, 8.7)	−2.5	(−4.8, −0.1)	*	8.2	(2.8, 13.7)	4.3	(0.8, 7.9)	
99–100	21.4, 25.4	18.6	(14.5, 22.7)	−4.6	(−7.4, −1.8)	**	26.6	(18.7, 34.4)	13.7	(9.2, 18.1)	**
Aged 65–75 years											
0–1	−33.4, −18.5	−1.8	(−6.4, 2.7)	0.9	(−3.0, 4.8)		−2.3	(−7.4, 2.8)	−1.2	(−9.8, 7.4)	
1–2.5	−18.5, −14.1	2.8	(−0.9, 6.4)	−3.6	(−6.6, −0.6)	*	5.9	(1.2, 10.6)	−1.2	(−7.1, 4.7)	
2.5–5	−14.1, −10.7	−0.4	(−3.2, 2.5)	−2.3	(−4.6, 0.0)		2.6	(−1.4, 6.5)	−4.1	(−8.3, 0.1)	*
5–10	−10.7, −6.6	−0.5	(−2.4, 1.5)	0.6	(−1.0, 2.1)		−0.8	(−3.4, 1.9)	−0.2	(−3.1, 2.8)	
10–25	−6.6, −0.8	1.7	(0.5, 2.8)	0.9	(0.0, 1.9)		0.6	(−1.1, 2.2)	2.7	(1.0, 4.4)	
25–50	−0.8, 4.8	−1.4	(−2.3, −0.5)	0.3	(−0.4, 1.0)		−1.3	(−2.5, −0.1)	−1.3	(−2.7, 0.1)	
50–75	4.8, 12.7	−1.4	(−2.3, −0.4)	−0.7	(−1.4, 0.1)		−0.9	(−2.2, 0.4)	−2.0	(−3.4, −0.6)	
75–90	12.7, 16.7	0.3	(−1.0, 1.5)	0.0	(−1.0, 0.9)		0.0	(−1.7, 1.7)	0.5	(−1.3, 2.3)	
90–95	16.7, 18.6	2.0	(−0.1, 4.29	−0.1	(−1.8, 1.6)		2.6	(−0.4, 5.6)	1.9	(−1.1, 5.0)	
95–97.5	18.6, 19.9	3.7	(0.6, 6.8)	1.1	(−1.3, 3.5)		3.1	(−0.8, 7.0)	4.2	(−0.5, 8.9)	
97.5–99	19.9, 21.4	4.3	(0.1, 8.4)	3.2	(−0.1, 6.59)		0.2	(−5.8, 6.1)	6.5	(0.9, 12.2)	
99–100	21.4, 25.4	14.4	(9.6, 19.3)	−5.9	(−9.2, −2.7)	***	24.7	(16.6, 32.8)	8.1	(2.3, 13.9)	**
Aged <65 years											
0–1	−33.4, −18.5	−3.0	(−7.3, 1.3)	0.7	(−2.9, 4.4)		−1.8	(−7.4, 3.8)	−4.9	(−11.7, 2.0)	
1–2.5	−18.5, −14.1	1.2	(−2.1, 4.6)	−0.1	(−2.8, 2.6)		0.6	(−3.7, 4.9)	1.2	(−4.1, 6.5)	
2.5–5	−14.1, −10.7	0.0	(−2.5, 2.5)	−0.5	(−2.5, 1.5)		0.8	(−2.2, 3.8)	−1.4	(−5.6, 2.8)	
5–10	−10.7, −6.6	1.3	(−0.5, 3.1)	0.3	(−1.1, 1.8)		1.6	(−0.8, 4.0)	1.1	(−1.7, 3.8)	
10–25	−6.6, −0.8	−0.1	(−1.1, 1.0)	−0.2	(−1.0, 0.6)		0.0	(−1.5, 1.4)	−0.4	(−1.9, 1.0)	
25–50	−0.8, 4.8	−0.3	(−1.1, 0.5)	0.1	(−0.6, 0.7)		−0.3	(−1.4, 0.8)	−0.2	(−1.4, 1.0)	
50–75	4.8, 12.7	−1.0	(−1.9, −0.2)	0.1	(−0.6, 0.7)		−1.0	(−2.2, 0.2)	−0.9	(−2.0, 0.3)	
75–90	12.7, 16.7	−0.9	(−1.9, 0.2)	−0.1	(−0.9, 0.8)		−1.1	(−2.6, 0.4)	−0.8	(−2.3, 0.7)	
90–95	16.7, 18.6	1.2	(−0.5, 3.0)	0.1	(−1.3, 1.4)		1.0	(−1.5, 3.5)	1.2	(−1.4, 3.7)	
95–97.5	18.6, 19.9	5.0	(2.1, 7.8)	−1.1	(−3.4, 1.1)		6.1	(2.2, 10.0)	3.5	(−0.6, 7.6)	
97.5–99	19.9, 21.4	7.1	(3.6, 10.5)	2.9	(0.2, 5.6)	*	3.3	(−2.1, 8.7)	9.2	(4.7, 13.6)	
99–100	21.4, 25.4	9.7	(5.7, 13.7)	−3.7	(−6.4, −1.0)	**	16.3	(10.2, 22.3)	5.6	(0.4, 10.8)	**

* *p* < 0.05, ** *p* < 0.01, *** *p* < 0.001.

## Supplementary Materials

The following are available online at www.mdpi.com/1660-4601/14/8/944/s1, Figure S1: Scatterplots of 7-day mean of relative mortality in different age groups and 7-day mean values of PET at Helsinki-Vantaa airport district in 1972–2014, and two 21-year time periods, 1972–1992 and 1994–2014 and their relationships fitted with the generalized additive model, GAM (95% CI). Figure S2: Scatterplots of 14-day means of relative mortality in different age groups and 14-day mean values of PET at Helsinki-Vantaa airport district in 1972–2014 and two 21-year time periods, 1972–1992 and 1994–014, and their relationships fitted with the generalized additive model, GAM (95% CI). Table S1: 7-day mean values of relative mortality (95% CI) of different age groups in the percentiles of 7-day mean values of PET at Helsinki-Vantaa airport in 1972–2014, and in two 21-year sub-periods, 1972–1992 and 1994–2014, and the statistical significance of the differences in relative mortality between the sub-periods. Table S2: 14-day mean values of relative mortality (95% CI) of different age groups in the percentiles of 14-day mean values of PET at Helsinki-Vantaa airport in 1972–2014, and in two 21-year sub-periods, 1972–1992 and 1994–2014, and the statistical significance of the differences in relative mortality between the sub-periods.
