# Supplementary material for: Biometeorological Assessment of Mortality Related to Extreme Temperatures in Helsinki Region, Finland, 1972–2014
Source: Int J Environ Res Public Health. 2017 Aug 22;14(8):944. doi: 10.3390/ijerph14080944 (PMC5580646; doi:10.3390/ijerph14080944)
Supplement: Supplementary file 1 [file ijerph-14-00944-s001.pdf]

Supplementary Materials: Biometeorological Assessment of Mortality from Extreme Temperatures in Helsinki Region, Finland, in 1972-2014

Reija Ruuhela, Kirsti Jylhä, Timo Lanki, Pekka Tiittanen and Andreas Matzarakis

Figure S1. Scatterplots of 7-day mean of relative mortality in different age groups and 7-day mean values of PET at Helsinki-Vantaa airport district in 1972–2014, and two 21-year time periods, 1972–1992 and 1994–2014 and their relationships fitted with the generalized additive model, GAM (95% CI).

Figure S2. Scatterplots of 14-day means of relative mortality in different age groups and 14-day mean values of PET at Helsinki-Vantaa airport district in 1972–2014 and two 21-year time periods, 1972–1992 and 1994–014, and their relationships fitted with the generalized additive model, GAM (95% CI).

Table S1. 7-day mean values of relative mortality (95% CI) of different age groups in the percentiles of 7-day mean values of PET at Helsinki-Vantaa airport in 1972–2014, and in two 21-year sub-periods, 1972–1992 and 1994–2014, and the statistical significance of the differences in relative mortality between the sub-periods.

Table S2. 14-day mean values of relative mortality (95% CI) of different age groups in the percentiles of 14-day mean values of PET at Helsinki-Vantaa airport in 1972–2014, and in two 21-year sub-periods, 1972–1992 and 1994–2014, and the statistical significance of the differences in relative mortality between the sub-periods.

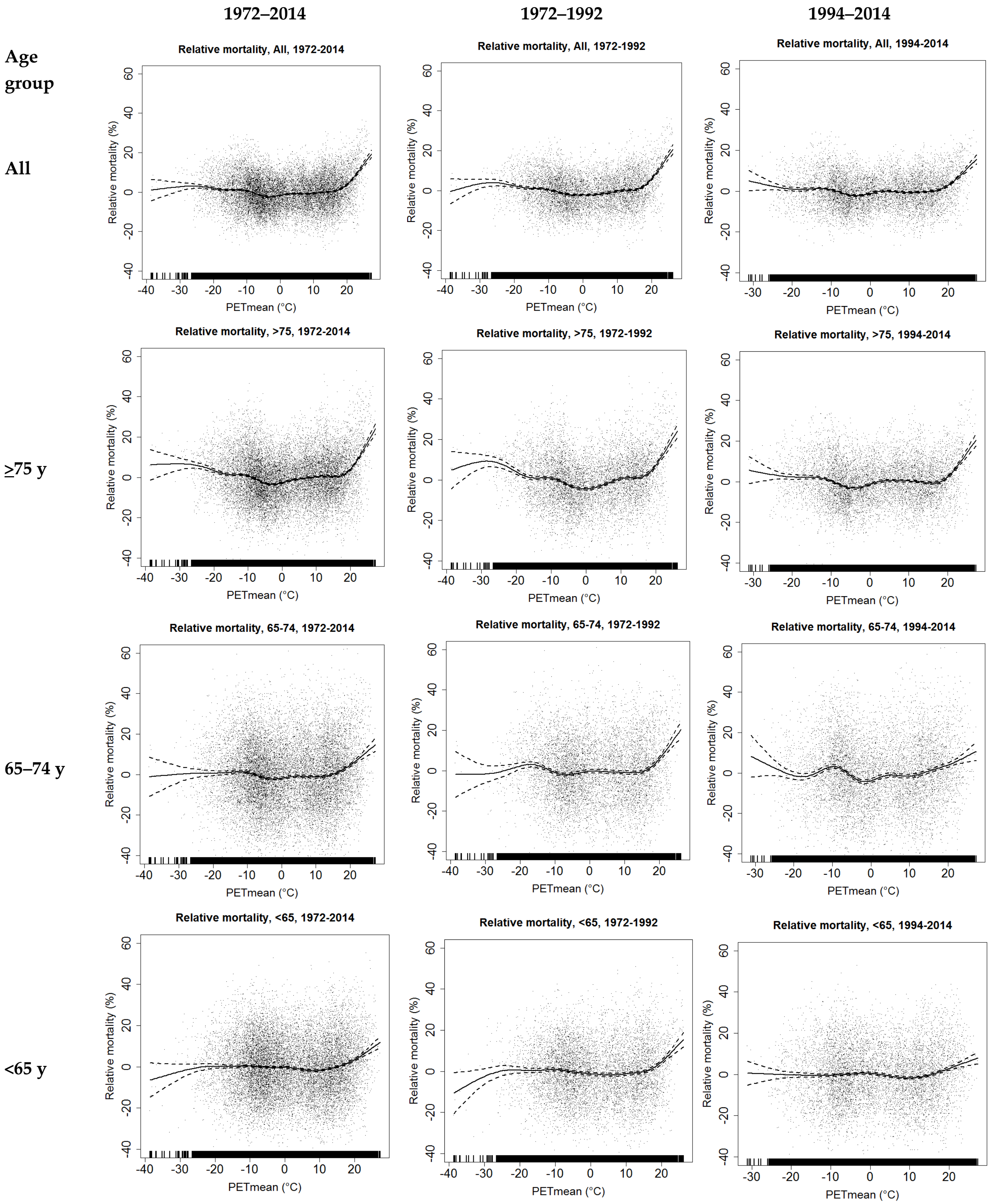

Figure S1. Scatterplots of 7-day mean of relative mortality in different age groups and 7-day mean values of PET at Helsinki-Vantaa airport district in 1972–2014, and two 21-year time periods, 1972–1992 and 1994–2014 and their relationships fitted with the generalized additive model, GAM (95% CI).

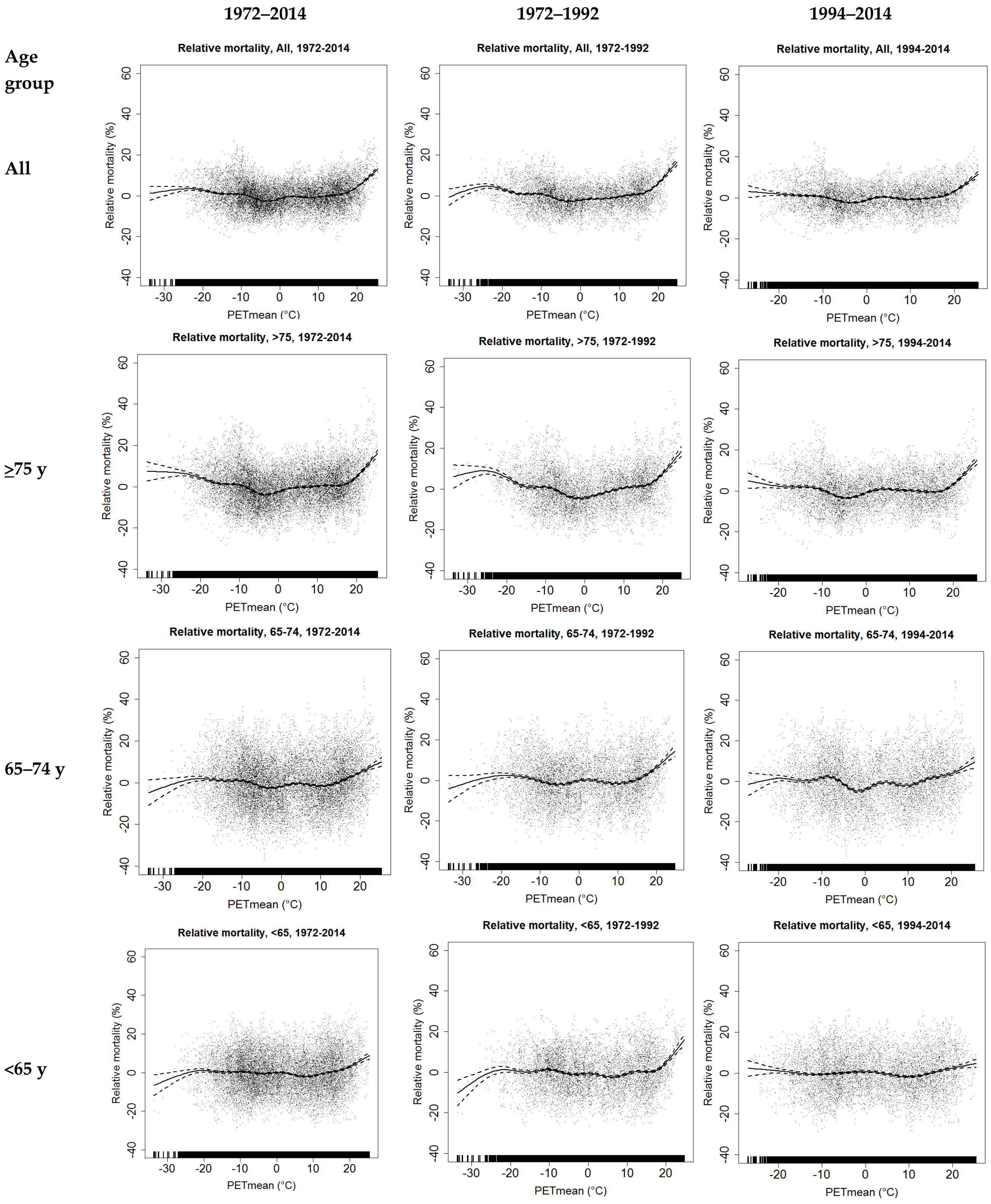

Figure S2. Scatterplots of 14-day means of relative mortality in different age groups and 14-day mean values of PET at Helsinki-Vantaa airport district in 1972–2014 and two 21-year time periods, 1972–1992 and 1994–014, and their relationships fitted with the generalized additive model, GAM (95% CI).

Table S1. 7-day mean values of relative mortality (95% CI) of different age groups in the percentiles of 7-day mean values of PET at Helsinki-Vantaa airport in 1972–2014, and in two 21-year sub-periods, 1972–1992 and 1994–2014, and the statistical significance of the differences in relative mortality between the sub-periods.* $p < 0.05$, ** $p < 0.01$, *** $p < 0.001$.

a) All

Percentiles	PETmean range		Relative Mortality 1972–2014 [%]			Relative Mortality 1972–1992 [%]			Relative Mortality 1994–2014 [%]			t-test
0–1	-38.7,	-22.2	1.7	(0.5,	2.9)	1.6	(0.2,	3.1)	1.8	(-0.3,	3.9)	
1–2.5	-22.2,	-18.9	2.9	(1.9,	3.9)	4.7	(3.4,	6.0)	1.1	(-0.3,	2.6)	***
2.5–5	-18.9,	-15.9	1.1	(0.2,	1.9)	1.6	(0.4,	2.8)	0.6	(-0.6,	1.7)	
5–10	-15.9,	-12.5	1.1	(0.4,	1.7)	0.8	(0.0,	1.6)	1.4	(0.4,	2.4)	
10–25	-12.5,	-7.4	0.5	(0.2,	0.8)	0.3	(-0.2,	0.8)	0.5	(0.1,	1.0)	
25–50	-7.4,	0.4	-1.9	(-2.1,	-1.6)	-1.7	(-2.1,	-1.4)	-2.0	(-2.3,	-1.6)	
50–75	0.4,	11.6	-0.9	(-1.1,	-0.6)	-1.3	(-1.7,	-1.0)	-0.4	(-0.8,	-0.1)	***
75–90	11.6,	16.8	0.3	(0.0,	0.7)	0.7	(0.3,	1.2)	-0.2	(-0.7,	0.3)	**
90–95	16.8,	19.4	1.7	(1.1,	2.2)	2.5	(1.7,	3.3)	1.0	(0.2,	1.7)	**
95–97.5	19.4,	21.3	4.1	(3.3,	4.9)	5.1	(3.7,	6.5)	3.5	(2.5,	4.5)	
97.5–99	21.3,	23.0	6.1	(4.8,	7.3)	7.9	(5.8,	10.0)	4.8	(3.3,	6.3)	*
99–100	23.0,	27.3	13.7	(11.8,	15.6)	21.5	(17.9,	25.2)	10.5	(8.5,	12.4)	***

b) Age ≥75 years

Percentiles	PETmean range		Relative Mortality 1972–2014 [%]			Relative Mortality 1972–1992 [%]			Relative Mortality 1994–2014 [%]			t-test
0–1	-38.7,	-22.2	4.3	(2.8,	5.9)	5.3	(3.5,	7.1)	2.9	(0.2,	5.7)	
1–2.5	-22.2,	-18.9	4.6	(3.3,	5.9)	6.7	(5.0,	8.4)	2.5	(0.7,	4.4)	**
2.5–5	-18.9,	-15.9	2.0	(1.0,	3.1)	2.4	(0.8,	4.0)	1.7	(0.3,	3.1)	
5–10	-15.9,	-12.5	1.5	(0.6,	2.3)	0.6	(-0.5,	1.8)	2.5	(1.2,	3.7)	*
10–25	-12.5,	-7.4	0.3	(-0.1,	0.8)	0.4	(-0.3,	1.1)	0.2	(-0.4,	0.9)	
25–50	-7.4,	0.4	-2.8	(-3.1,	-2.5)	-2.9	(-3.4,	-2.4)	-2.8	(-3.2,	-2.3)	
50–75	0.4,	11.6	-0.5	(-0.9,	-0.2)	-1.5	(-2.0,	-0.9)	0.2	(-0.2,	0.7)	***
75–90	11.6,	16.8	0.6	(0.2,	1.1)	1.7	(1.0,	2.3)	-0.3	(-0.9,	0.3)	***
90–95	16.8,	19.4	1.6	(0.8,	2.4)	3.4	(2.2,	4.5)	0.1	(-0.8,	1.1)	***
95–97.5	19.4,	21.3	4.7	(3.5,	6.0)	7.3	(4.9,	9.7)	3.2	(1.7,	4.6)	**
97.5–99	21.3,	23.0	6.7	(4.7,	8.7)	10.5	(6.6,	14.3)	4.2	(2.2,	6.2)	**
99–100	23.0,	27.3	16.7	(14.2,	19.1)	23.5	(19.0,	28.1)	13.8	(11.1,	16.6)	***

c) Age 65–74 years

Percentiles	PETmean range		Relative Mortality 1972–2014 [%]			Relative Mortality 1972–1992 [%]			Relative Mortality 1994–2014 [%]			t-test
0–1	-38.7,	-22.2	-0.6	(-2.9,	1.7)	-2.0	(-4.9,	0.8)	1.4	(-2.5,	5.2)	
1–2.5	-22.2,	-18.9	2.3	(0.2,	4.3)	5.3	(2.7,	7.8)	-0.7	(-3.8,	2.5)	**
2.5–5	-18.9,	-15.9	-0.3	(-1.8,	1.3)	2.3	(0.1,	4.6)	-2.9	(-5.1,	-0.7)	**
5–10	-15.9,	-12.5	1.5	(0.5,	2.6)	2.4	(0.8,	3.9)	0.7	(-0.9,	2.3)	
10–25	-12.5,	-7.4	1.1	(0.5,	1.7)	-1.0	(-1.8,	-0.1)	3.1	(2.2,	4.0)	***
25–50	-7.4,	0.4	-1.9	(-2.4,	-1.4)	-1.2	(-1.8,	-0.6)	-2.5	(-3.2,	-1.7)	*
50–75	0.4,	11.6	-1.2	(-1.7,	-0.7)	-0.9	(-1.6,	-0.2)	-1.6	(-2.3,	-0.9)	
75–90	11.6,	16.8	0.2	(-0.5,	0.9)	-0.2	(-1.1,	0.7)	0.5	(-0.5,	1.5)	
90–95	16.8,	19.4	2.7	(1.6,	3.8)	2.8	(1.2,	4.4)	2.7	(1.1,	4.3)	
95–97.5	19.4,	21.3	4.6	(3.0,	6.2)	5.4	(2.6,	8.1)	4.2	(2.1,	6.2)	
97.5–99	21.3,	23.0	6.3	(4.1,	8.5)	8.1	(5.2,	11.0)	5.0	(1.9,	8.2)	
99–100	23.0,	27.3	11.8	(9.0,	14.5)	20.0	(14.6,	25.5)	8.4	(5.4,	11.4)	***

d) Age <65 years

Percentiles	PETmean range		Relative Mortality 1972–2014 [%]			Relative Mortality 1972–1992 [%]			Relative Mortality 1994–2014 [%]			t-test
0–1	-38.7,	-22.2	-1.0	(-2.9,	0.9)	-1.5	(-4.2,	1.3)	-0.4	(-3.0,	2.1)	
1–2.5	-22.2,	-18.9	0.7	(-0.9,	2.4)	2.0	(-0.2,	4.2)	-0.5	(-2.9,	2.0)	
2.5–5	-18.9,	-15.9	0.3	(-1.0,	1.6)	0.2	(-1.6,	2.0)	0.4	(-1.5,	2.4)	
5–10	-15.9,	-12.5	-0.1	(-1.1,	0.9)	0.2	(-1.1,	1.4)	-0.7	(-2.3,	0.9)	
10–25	-12.5,	-7.4	0.3	(-0.2,	0.9)	0.9	(0.2,	1.7)	-0.6	(-1.4,	0.2)	**
25–50	-7.4,	0.4	-0.2	(-0.6,	0.2)	-0.5	(-1.1,	0.1)	0.3	(-0.4,	0.9)	
50–75	0.4,	11.6	-1.5	(-1.9,	-1.0)	-1.6	(-2.2,	-1.0)	-1.0	(-1.6,	-0.5)	
75–90	11.6,	16.8	0.1	(-0.5,	0.7)	0.1	(-0.7,	1.0)	-0.5	(-1.3,	0.4)	
90–95	16.8,	19.4	1.2	(0.2,	2.3)	1.2	(-0.2,	2.6)	1.3	(-0.3,	2.8)	
95–97.5	19.4,	21.3	3.4	(2.1,	4.8)	2.3	(0.2,	4.4)	4.1	(2.3,	5.8)	
97.5–99	21.3,	23.0	5.6	(3.8,	7.4)	5.6	(2.9,	8.2)	5.6	(3.2,	8.0)	
99–100	23.0,	27.3	8.6	(6.2,	10.9)	19.9	(15.4,	24.4)	4.0	(1.7,	6.3)	***

Table S2. 14-day mean values of relative mortality (95% CI) of different age groups in the percentiles of 14-day mean values of PET at Helsinki-Vantaa airport in 1972–2014, and in two 21-year sub-periods, 1972–1992 and 1994–2014, and the statistical significance of the differences in relative mortality between the sub-periods.
* $p < 0.05$, ** $p < 0.01$, *** $p < 0.001$.

a) All

Percentiles	PETmean range		Relative Mortality 1972–2014 [%]			Relative Mortality 1972–1992 [%]			Relative Mortality 1994–2014 [%]			t-test
0–1	-33.7,	-20.8	2.7	(1.7,	3.6)	2.8	(1.9,	3.8)	2.3	(-0.2,	4.7)	
1–2.5	-20.8,	-17.7	2.5	(1.7,	3.3)	3.6	(2.4,	4.8)	1.4	(0.3,	2.6)	*
2.5–5	-17.7,	-15.5	1.9	(1.2,	2.5)	1.7	(0.8,	2.7)	2.1	(1.2,	2.9)	
5–10	-15.5,	-12.4	0.7	(0.2,	1.2)	1.0	(0.3,	1.6)	0.4	(-0.3,	1.0)	
10–25	-12.4,	-7.6	0.6	(0.3,	0.8)	0.4	(0.0,	0.8)	0.5	80.1,	0.9)	
25–50	-7.6,	0.4	-1.9	(-2.1,	-1.8)	-2.0	(-2.2,	-1.8)	-1.8	(-2.1,	-1.6)	
50–75	0.4,	11.6	-0.7	(-0.9,	-0.5)	-1.1	(-1.3,	-0.8)	-0.4	(-0.6,	-0.1)	***
75–90	11.6,	16.6	0.5	(0.3,	0.8)	1.1	(0.7,	1.4)	-0.1	(-0.4,	0.3)	***
90–95	16.6,	18.8	1.7	(1.3,	2.1)	2.1	(1.5,	2.7)	1.4	(0.8,	1.9)	
95–97.5	18.8,	20.5	3.4	(2.8,	4.1)	5.6	(4.7,	6.6)	2.0	(1.2,	2.8)	***
97.5–99	20.5,	22.0	4.2	(3.3,	5.1)	4.1	(2.2,	6.1)	4.3	(3.2,	5.3)	
99–100	22.0,	25.4	12.0	(10.8,	13.1)	16.8	(14.8,	18.8)	9.4	(8.2,	10.5)	***

b) Age ≥75 years

Percentiles	PETmean range		Relative Mortality 1972–2014 [%]			Relative Mortality 1972–1992 [%]			Relative Mortality 1994–2014 [%]			t-test
0–1	-33.7,	-20.8	5.3	(4.0,	6.6)	6.4	(5.3,	7.6)	2.8	(-0.4,	6.1)	*
1–2.5	-20.8,	-17.7	3.4	(2.3,	4.4)	5.0	(3.5,	6.4)	1.8	(0.3,	3.2)	**
2.5–5	-17.7,	-15.5	3.1	(2.3,	3.8)	2.9	(1.8,	4.0)	3.2	(2.1,	4.3)	
5–10	-15.5,	-12.4	1.2	(0.6,	1.8)	1.4	(0.4,	2.3)	1.0	(0.2,	1.9)	
10–25	-12.4,	-7.6	0.5	(0.1,	0.9)	0.3	(-0.3,	0.8)	0.6	(0.1,	1.2)	
25–50	-7.6,	0.4	-2.8	(-3.0,	-2.6)	-2.9	(-3.3,	-2.6)	-2.7	(-3.0,	-2.4)	
50–75	0.4,	11.6	-0.4	(-0.6,	-0.1)	-1.3	(-1.6,	-0.9)	0.4	(0.1,	0.7)	***
75–90	11.6,	16.6	0.9	(0.6,	1.2)	2.2	(1.7,	2.6)	-0.4	(-0.8,	0.0)	***
90–95	16.6,	18.8	1.5	(1.0,	2.1)	2.6	(1.7,	3.5)	0.7	(0.0,	1.5)	**
95–97.5	18.8,	20.5	3.8	(2.9,	4.7)	6.6	(4.8,	8.4)	2.0	(1.0,	3.0)	***
97.5–99	20.5,	22.0	4.4	(3.2,	5.6)	4.9	(2.2,	7.6)	4.2	(2.9,	5.6)	
99–100	22.0,	25.4	14.5	(12.6,	16.4)	19.9	(16.3,	23.5)	11.5	(9.5,	13.5)	***

c) Age 65–74 years

Percentiles	PETmean range		Relative Mortality 1972–2014 [%]			Relative Mortality 1972–1992 [%]			Relative Mortality 1994–2014 [%]			t-test
0–1	-33.7,	-20.8	0.6	(-0.6,	1.8)	0.4	(-1.1,	1.9)	1.0	(-0.9,	3.0)	
1–2.5	-20.8,	-17.7	1.9	(0.5,	3.3)	3.4	(1.5,	5.3)	0.4	(-1.6,	2.4)	*
2.5–5	-17.7,	-15.5	2.3	(1.1,	3.5)	2.6	(1.0,	4.2)	2.0	(0.3,	3.7)	
5–10	-15.5,	-12.4	0.2	(-0.6,	1.0)	1.0	(-0.1,	2.2)	-0.8	(-1.8,	0.3)	*
10–25	-12.4,	-7.6	0.8	(0.4,	1.2)	-0.2	(-0.8,	0.4)	1.8	(1.1,	2.5)	***
25–50	-7.6,	0.4	-1.8	(-2.1,	-1.5)	-1.7	(-2.1,	-1.3)	-1.9	(-2.4,	-1.3)	
50–75	0.4,	11.6	-1.0	(-1.4,	-0.7)	-0.5	(-0.9,	0.0)	-1.7	(-2.2,	-1.2)	***
75–90	11.6,	16.6	0.5	(0.0,	0.9)	0.1	(-0.5,	0.7)	1.0	(0.3,	1.6)	*
90–95	16.6,	18.8	2.6	(1.8,	3.3)	2.4	(1.3,	3.6)	2.7	(1.7,	3.8)	
95–97.5	18.8,	20.5	4.1	(3.0,	5.2)	7.1	(5.7,	8.6)	2.1	(0.6,	3.5)	***
97.5–99	20.5,	22.0	4.8	(3.2,	6.4)	3.9	(1.5,	6.3)	5.1	(3.1,	7.2)	
99–100	22.0,	25.4	10.8	(9.1,	12.5)	14.8	(12.2,	17.4)	8.6	(6.5,	10.7)	***

d) Age <65 years

Percentiles	PETmean range		Relative Mortality 1972–2014 [%]			Relative Mortality 1972–1992 [%]			Relative Mortality 1994–2014 [%]			t-test
0–1	-33.7,	-20.8	-0.3	(-1.7,	1.1)	-1.2	(-2.9,	0.5)	1.8	(1.8,	2.5)	*
1–2.5	-20.8,	-17.7	2.0	(0.8,	3.2)	2.3	(0.5,	4.2)	1.7	(1.7,	1.5)	
2.5–5	-17.7,	-15.5	-0.2	(-1.2,	0.7)	-0.1	(-1.6,	1.3)	-0.4	-0.4,	1.2)	
5–10	-15.5,	-12.4	0.3	(-0.4,	0.9)	0.5	(-0.4,	1.4)	-0.2	(-0.2,	0.9)	
10–25	-12.4,	-7.6	0.3	(-0.1,	0.7)	1.0	(0.5,	1.5)	-0.7	(-0.7,	0.6)	***
25–50	-7.6,	0.4	-0.4	(-0.6,	-0.1)	-0.8	(-1.2,	-0.4)	0.3	(0.3,	0.4)	***
50–75	0.4,	11.6	-1.4	(-1.7,	-1.1)	-1.5	(-1.9,	-1.1)	-1.1	(-1.1,	0.4)	
75–90	11.6,	16.6	0.4	(-0.1,	0.8)	0.4	(-0.2,	1.0)	-0.1	(-0.1,	0.6)	
90–95	16.6,	18.8	1.3	(0.6,	2.0)	1.0	(0.1,	1.9)	1.5	(1.5,	1.0)	
95–97.5	18.8,	20.5	2.7	(1.6,	3.7)	3.5	(2.1,	5.0)	2.1	(2.1,	1.4)	
97.5–99	20.5,	22.0	3.5	(2.2,	4.8)	3.3	(0.7,	5.9)	3.6	(3.6,	1.5)	
99–100	22.0,	25.4	8.2	(6.9,	9.6)	15.5	(14.0,	17.1)	4.3	(4.3,	1.4)	***
